# Association of adult lung function with accelerated biological aging

**DOI:** 10.18632/aging.102639

**Published:** 2020-01-11

**Authors:** Faisal I. Rezwan, Medea Imboden, Andre F.S. Amaral, Matthias Wielscher, Ayoung Jeong, Kai Triebner, Francisco Gómez Real, Marjo-Riitta Jarvelin, Deborah Jarvis, Nicole M. Probst-Hensch, John W. Holloway

**Affiliations:** 1 Human Development and Health, Faculty of Medicine, University of Southampton, Southampton, United Kingdom; 2 Chronic Disease Epidemiology Unit, Department of Epidemiology and Public Health, Swiss Tropical and Public Health Institute, Basel, Switzerland; 3 University of Basel, Basel, Switzerland; 4 Population Health and Occupational Disease, NHLI, Imperial College London, London, United Kingdom; 5 MRC-PHE Centre for Environment and Health, Imperial College London, London, United Kingdom; 6 Department of Epidemiology and Biostatistics, MRC–PHE Centre for Environment and Health, School of Public Health, Imperial College London, London, United Kingdom; 7 Department of Clinical Science, University of Bergen, Bergen, Norway; 8 Department of Gynecology and Obstetrics, University of Bergen, Bergen, Norway

**Keywords:** respiratory health, lung function, epigenetic clock, DNA methylation, age acceleration

## Abstract

Lung function, strongly associated with morbidity and mortality, decreases with age. This study examines whether poor adult lung function is associated with age accelerations (AAs). DNA methylation (DNAm) based AAs, lifespan predictors (GrimAge and plasminogen activator inhibitor 1-PAI1) and their related age-adjusted measures were estimated from peripheral blood at two time points (8-to-11 years apart) in adults from two cohorts: SAPALDIA (n=987) and ECRHS (n=509). Within each cohort and stratified by gender (except for estimators from GrimAge and PAI1), AAs were used as predictors in multivariate linear regression with cross-sectional lung function parameters, and in covariate-adjusted mixed linear regression with longitudinal change in lung function and meta-analysed.

AAs were found cross-sectionally associated with lower mean FEV1 (Forced Expiratory Volume in one second) (AA-residuals:P-value=4x10^-4^; Intrinsic Epigenetic AA:P-value=2x10^-4^) in females at the follow-up time point only, and the same trend was observed for FVC (Forced Vital Capacity). Both lifespan and plasma level predictors were observed strongly associated with lung function decline and the decline was stronger in the follow-up time points (strongest association between FEV1 and DNAmAge GrimAge:P-value=1.25x10^-17^).

This study suggests that DNAm based lifespan and plasma level predictors can be utilised as important factors to assess lung health in adults.

## INTRODUCTION

Lung function is an important predictor of mortality [1] even in non-smokers, with low adult lung function being a consequence of poor growth *in utero* and/or childhood and/or excessive decline in adult life [2]. Lung function decline in adulthood occurs because of anatomical, physiological and immunological age-related changes in the lung [3], with the rate of change influenced by both genetics [4] and environmental exposures including smoking, occupational exposures and air pollution [5–7]. However, the exact mechanisms contributing to lung function decline are not fully understood.

Clinicians and members of the public have long noted that some individuals have considerable mismatch between their chronological age and their apparent biological age. There are now methods to formally quantify biological age using biospecimens and one of the most widely reported and examined is ‘epigenetic aging’ based on peripheral blood DNA methylation (DNAm).

There are several methods available to estimate epigenetic aging [8–12] and both the Horvath and Hannum methods for epigenetic age estimation (DNAmAge) have shown high accuracy, with an average correlation > 0.90 between chronological and epigenetic age [10]. However, these correlations are heterogeneous with the Horvath and Hannum methods demonstrating a median absolute difference between DNAmAge and chronological age of 3.5 [10] and 4.9 years [9], respectively. The difference between epigenetic age and chronological age is known as age acceleration (AA) and both epigenetic age measures and AAs are highly correlated with the chronological age. Therefore, residuals from regression between epigenetic and chronological ages (AA_res_), using Horvath method, are used to determine epigenetic age acceleration. In addition, the AA measures are confounded by age-related functional decline in blood cell composition. Therefore, intrinsic epigenetic age acceleration (IEAA) is used, which is independent of age related changes of cellular composition of blood, contrasting extrinsic epigenetic age acceleration (EEAA), incorporating age-related changes in cellular composition in blood and intrinsic epigenetic changes [13]. Most recently, DNAm GrimAge (DNAmAge_grim_), a predictor of lifespan, has been developed based on seven DNAm surrogates and a DNAm-based estimator of smoking pack-years. The age acceleration, known as AgeAccelGrim, can also be determined from DNAm GrimAge and henceforth will be denoted as AA_grim_ [14]. In addition, a DNA methylation-based surrogate of plasma protein namely plasminogen activator inhibitor level (DNAmPAI1) and its age adjusted estimator (DNAmPAI1_adj_), developed in the same study, can be good biomarkers of aging. Several recent studies, using the Horvath and Hannum methods, have found age acceleration is associated with a number of diseases and phenotypes, such as obesity [15], Alzheimer’s disease [16], Down’s syndrome [17], Huntington disease [18], HIV [19], Parkinson’s disease [20], and earlier menopause [21]. Horvath’s ‘epigenetic clock’ has also been found to be associated with mortality. For example in a study of older people (> 68 years), those with an apparent epigenetic age 5 years greater than their chronological age had a 21% increased mortality risk over the following 5 years when compared to those with no evidence of age acceleration [22]. DNAmAge_grim_ has been found to be a superior predictor of time-to-death and DNAmPAI1 has been observed to be associated with lifespan, comorbidity count and type 2 diabetes [14].

To date little is known regarding the association of epigenetic aging, as measured from peripheral blood, and lung function. The 1936 Mid-Lothian Birth Cohort examined the association of various physical measures with epigenetic aging in over 1000 elderly adults (mean age of 69 ± 0.83 years) followed for between 3 and 6 years. Lung function, considered as FEV_1_ (forced expiratory volume in one second), was the only one of four physiological measures of aging (others being cognition, grip strength and walking speed) to show an association with DNAmAge, albeit statistically weak (P-value = 0.05), and small in effect size (<1 mL change in FEV_1_ per additional year of epigenetic aging). Epigenetic aging explained only 0.33% of the variance in FEV_1_ decline [23].

As part of the aging Lungs in European Cohorts (ALEC) study (www.alecstudy.org) we obtained DNA methylation information from 1,496 adults (age range at baseline: 37 to 61 years), followed for 8 to 11 years, derived from two population-based cohorts specifically designed to investigate lung function. The aim of our study was to examine the cross-sectional and longitudinal association of peripheral blood epigenetic signature of aging with lung function in these general population-based samples of adults using data on both lung function and epigenetic age at two time points multiple years apart.

## RESULTS

Descriptive statistics of the cohorts at baseline and follow-up time points are presented in Table 1. The time intervals between the two lung function assessments in Swiss study of Air Pollution and Lung and heart Disease in Adults (SAPALDIA) and the European Community Respiratory Health Survey (ECRHS) were 8.3 and 10.9 years respectively. The SAPALDIA cohort were older with a wider range of ages than the ECRHS (baseline: 50.55 ± 11.3 vs. 43.64 ± 6.76 and follow-up: 58.85 ± 11.26 vs. 54.54 ± 6.78 years). Each cohort had similar proportions of men and women.

**Table 1:** Summary of the variables for cohort-specific study populations.

		**Baseline**		**Follow-up**	
		**SAPALDIA**	**ECRHS**	**SAPALDIA**	**ECRHS**
N		987	509	987	509
Age (years)		50.55 ± 11.3	43.64 ± 6.76	58.85 ± 11.26	54.54 ± 6.78
Female (%)		528 (53.50)	290 (56.98)	Same as baseline	Same as baseline
BMI (kg/m2)		25.8 ± 4.38	25.23 ± 4.25	26.47 ± 4.61	26.73 ± 4.56
Height (cm)		169.49 ± 9.27	169.59 ± 9.35	168.77 ± 9.4	168.89 ± 9.35
Smoking	Never	407 (41.24)	216(42.44)	401 (40.63)	208(40.87)
	Ex	297 (30.09)	165(32.41)	366 (37.08)	209(41.06)
	Current	282 (28.57)	128(25.15)	220 (22.29)	92(18.07)
Pack years		11.95 ± 18.36	9.28 ± 14.89	13.36 ± 20.19	14.02 ± 32.08
Education^†^	1	54 (5.48)	66 (12.97)	Same as baseline	Same as baseline
	2	644 (65.25)	148 (29.08)	Same as baseline	Same as baseline
	3	288 (29.18)	295 (57.96)	Same as baseline	Same as baseline
Ever asthma		124 (12.56)	79 (15.52)	111 (11.25)	95 (18.66)
FEV_1_ (L)		3.25 ± 0.83	3.41 ± 0.78	2.96 ± 0.84	2.95 ± 0.75
FVC (L)		4.35 ± 1.05	4.25 ± 0.97	4.05 ± 1.06	3.92 ± 0.97
FEV_1_/FVC		0.75 ± 0.07	0.8 ± 0.06	0.73 ± 0.08	0.75 ± 0.06

Data are presented as n (%) for categorical and mean ± SD for continuous variables.

† For SAPALDIA: 1: Low (primary school); 2: Middle (secondary school, middle school or apprenticeship); 3: High (Technical College or University). For ECRHS: education finishes at 1: ≤16 year; 2: 17-19 year; 3: 20+ years.

Within SAPALDIA, chronological age was more highly correlated with DNAm Age (baseline = 0.91; follow-up = 0.89) than in the ECRHS (baseline = 0.64; follow-up = 0.71). However, the median absolute deviation suggested little variability and the probability of outliers in estimated DNAmAge was low (Table 2).

**Table 2:** Summary of chronological and DNAmAge derived from methylation values presented as mean ± SD.

	**N**	**Age (years)**	**DNAmAge**	**R**	**MAD**
SAPALDIA (baseline)	987	50.55 ± 11.3	52.07± 10	0.91	3.4
SAPALDIA (follow-up)	987	58.85± 11.26	58.5 ± 9.78	0.89	3.3
ECRHS (baseline)	509	43.64 ± 6.76	47.04 ± 8.32	0.64	3.8
ECRHS (follow-up)	509	54.54 ± 6.78	55.75 ± 7.43	0.71	2.9

R = correlation between chronological and DNAmAge. Here DNAmAge has been calculated using Horvath method. MAD = Median Absolute Deviation.

### Cross-sectional association between lung function and age acceleration at baseline and follow-up time point separately

**Figure 1:**
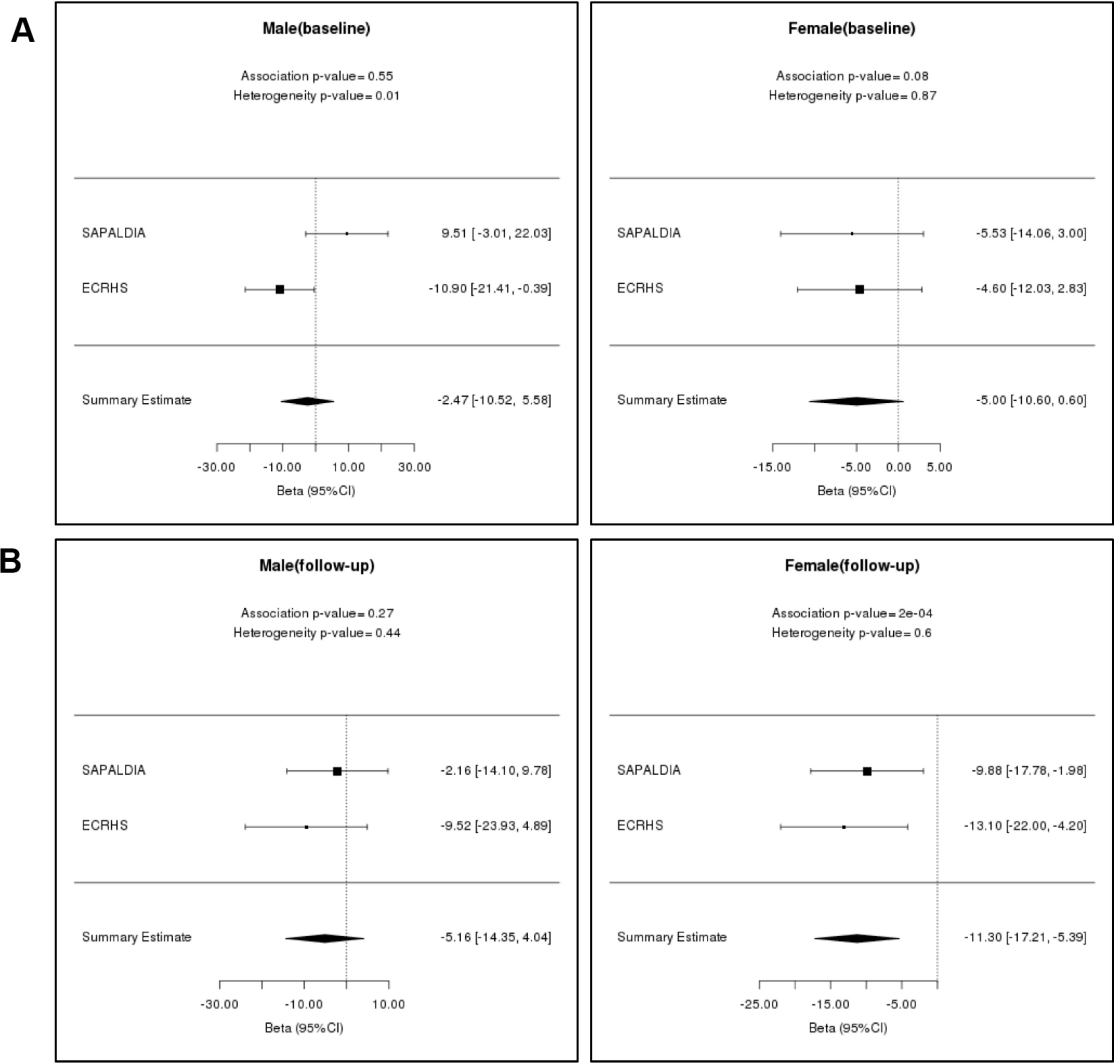
**Cross-sectional meta-analysis results for FEV_1_ of males and females in SAPALDIA and ECRHS.** (**A**) Mean change in FEV_1_ (mL) per year of intrinsic epigenetic age acceleration at baseline; (**B**) Mean change in FEV_1_ (mL) per year of intrinsic epigenetic age acceleration at follow-up. The estimates have been measured with 95% confidence interval in mL/year.

Results from linear models examining associations of forced expiratory volume in one second (FEV_1_) with age acceleration at each time point cross-sectionally within SAPALDIA and ECRHS are presented in Table 3. Effect estimates were larger in women than men and reached statistical significance (P-value < 0.05) at follow-up time point in women only. In women, at the follow-up time point, FEV_1_ was associated with AA_res_ (P-value = 4 x 10^-4^), where with one year increase of AA_res_, there was a decrement of 9.52 mL in FEV_1_ (CI: -14.77 mL/year_AA_ to −4.28 mL/year_AA_,). The same was been observed for IEAA, where FEV_1_ was 11.30 mL lower per year increase of IEAA (95% CI: -17.21 mL/year_IEAA_ to −4.20 mL/year_IEAA_ and P-value = 2 x 10^-4^). A marginal association between EEAA and FEV_1_ was observed in the same time point for female subjects (estimate = -5.11 mL/year_EEAA_; 95% CI: -10.16 mL/year_EEAA_ to 0.01 mL/year_EEAA_, P-value = 0.05). (Table 3, Figure 1). EEAA was found marginally significantly associated (P-value = 0.05) with only FEV_1_, in women at the later time point.

**Table 3:** Cross-sectional meta-analysis results of association between age acceleration and FEV_1_ and FVC in SAPALDIA and ECRHS cohorts.

**Lung functions**	**Sex**	**Age acceleration**	**Baseline**				**Follow-up**			
			**Estimate**	**Lower bound**	**Upper bound**	**P-value**	**Estimate**	**Lower bound**	**Upper bound**	**P-value**
FEV_1_	Male	AA_res_	-1.27	−9.16	6.62	0.75	-5.39	−14.48	3.69	0.25
		IEAA	-2.47	-10.52	5.58	0.55	-5.16	-14.35	4.04	0.44
		EEAA	-0.94	-8.44	6.55	0.81	-6.32	-15.24	2.59	0.17
	Female	AA_res_	-3.02	-9.17	1.34	0.14	-9.52	−14.77	−4.28	4 x 10^-04^*
		IEAA	-5.00	-10.60	0.60	0.08	-11.30	-17.21	-4.20	2 x 10^-04^*
		EEAA	-4.35	-9.39	0.69	0.09	-5.107	-10.16	0.01	0.05*
FVC	Male	AA_res_	-4.25	-13.31	4.82	0.36	-10.83	-20.95	-0.71	0.04*
		IEAA	-5.04	-14.30	4.22	0.30	-9.29	-19.57	0.99	0.08
		EEAA	0.69	-7.85	9.23	0.87	-4.69	-10.46	1.26	0.12
	Female	AA_res_	-4.61	-10.64	1.42	0.13	-9.31	-15.42	-3.20	0.003*
		IEAA	-5.21	-11.65	1.23	0.11	-10.49	-17.37	-3.60	0.003*
		EEAA	-8.06	-17.90	1.78	0.11	-4.86	-10.76	1.03	0.11

Here, Estimate = difference in lung function per year of epigenetic age acceleration (mL/year). Negative values denote that with every year of increase in epigenetic age acceleration, FEV_1_ decreases and vice-versa; Lower and upper = lower and upper ranges of 95% confidence interval of estimates; P-value = p-values from meta-analyses.

A similar association for women at the later time point was found for forced vital capacity (FVC) (AA_res_: -9.31 mL/year; 95% CI: -15.42 mL/year to −3.20 mL/year, P-value = 0.003 and IEAA: -10.49 mL/year; 95% CI: -17.37 mL/year to −3.60 mL/year, P-value = 0.003) (Table 3) and for FEV_1_/FVC, IEAA was found associated (Supplementary Table 1C).

In men, only AA_res_ was found to be significantly associated with FVC (-10.83 mL/year; 95% CI: -20.95 mL/year to −0.71 mL/year, P-value = 0.04) from the follow-up.

### Association between lung function and age acceleration from repeated measures at baseline and follow-up time points combined

**Figure 2:**
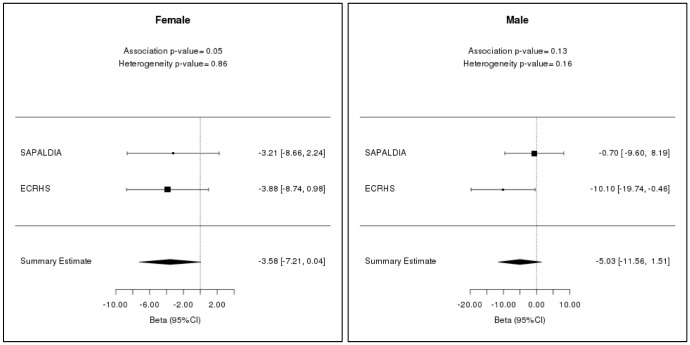
**Linear mixed model meta-analysis results for FEV_1_ of males and females in SAPALDIA and ECRHS for two time points (baseline and follow-up).** The estimates have been measured with 95% confidence interval in mL/year.

In women, there was evidence for a weak association of lower FEV_1_ with EEAA (estimate = -3.58 mL/year; 95% CI: -7.21 mL/year to 0.04 mL/year and P-value = 0.05) (Table 4; Figure 2). There was no evidence that age acceleration is associated with lung function in men (Supplementary Table 2A).

**Table 4:** Meta-analysis results of repeat cross-sectional association between age acceleration and FEV_1_ in SAPALDIA and ECRHS cohorts from two time points (baseline and follow-up).

	**Age acceleration**	**Estimate**	**Lower bound**	**Upper bound**	**P-value**
Male	AA_res_	1.20	-3.41	5.81	0.87
	IEAA	3.23	-1.21	7.68	0.9
	EEAA	-5.03	-11.56	1.51	0.13
Female	AA_res_	-1.56	-4.10	0.99	0.13
	IEAA	-1.38	-4.23	1.47	0.19
	EEAA	-3.58	-7.21	0.04	0.05

Here, Estimate = changes in lung function per year of epigenetic age acceleration (mL/year). Negative values denote that with every unit of increase in epigenetic age acceleration, FEV_1_ decreases and vice-versa; Lower and upper = lower and upper ranges of 95% confidence interval of estimates; P-value = p-values from meta-analyses.

### Association between longitudinal change in epigenetic age acceleration and change in lung function changes over follow-up

A linear model was used to investigate whether the change in biological aging between baseline and follow-up was associated with rate of change in lung function between the two time points. Both cohorts showed no association of lung function decline with change in biological age acceleration.

A weak association of rate of FEV_1_ change with biological age acceleration derived from IEAA (0.52 mL/year of epigenetic age acceleration; 95% CI: -0.02 mL/epigenetic year to 1.05 mL/ epigenetic year and P-value = 0.06) was observed in men (Supplementary Table 3). This same trend was not seen in women (0.18 mL/year of epigenetic age; 95% CI: -0.15 mL/epigenetic year to 0.05 mL/ epigenetic year and P-value = 0.2).

### Effect of menopause on lung function and epigenetic age acceleration

We incorporated a variable indicating menopausal status (pre-, peri-, and post-menopausal) at follow-up for the 528 SAPALDIA and 223 ECRHS women. AA_res_ and IEAA at follow-up remained associated with FEV_1_ at follow-up in women (AA_res_: -9.99 mL/year; 95% CI: -16.03 mL/year to −3.96 mL/year and P-value = 0.001 and IEAA: -10.81 mL/year; 95% CI: -17.12 mL/year to −4.49 mL/year and P-value = 0.001). However, the effect size of FEV_1_ for female samples for the follow-up time point was marginally reduced. The association between FVC, and AA_res_ and IEAA also remained significant following adjustment for menopausal status (Table 5). Comparison of the meta-analyses with and without menopausal status using ANOVA showed no significant differences (Supplementary Table 4).

**Table 5:** Meta-analysis results of repeat cross-sectional association between age acceleration and FEV_1_ in SAPALDIA and ECRHS cohorts from two time points (baseline and follow-up) in women (SAPALDIA: n=528; ECRHS: n=290), adjusted for menopausal status.

**Lung functions**	**Age acceleration**	**Estimate**	**Lower estimate**	**Upper estimate**	**P-value**
FEV_1_	AA_res_	-9.99	-16.03	-3.96	0.001*
	IEAA	-10.81	-17.12	-4.49	0.001*
	EEAA	-5.21	-10.51	0.1	0.06
FVC	AA_res_	-10.23	-17.25	-3.22	0.004*
	IEAA	-10.97	-18.32	-3.63	0.003*
	EEAA	-5.86	-12.00	0.28	0.06
FEV_1_/FVC	AA_res_	-0.0008	-0.002	0.0002	0.108
	IEAA	-0.0008	-0.001	0.0003	0.138
	EEAA	-0.0007	-0.002	0.0005	0.255

Estimate = difference in lung function per year of epigenetic age acceleration (mL/year for FEV_1_ and FVC). Negative values denote that with every unit of increase in epigenetic age acceleration lung function decreases and vice-versa; Lower and upper = lower and upper ranges of 95% confidence interval of estimates; P-value = p-values from meta-analysis.

The stratified cross-sectional analysis of female samples showed association of marginal significance (P-value = 0.057) in lung function (FEV_1_) decline (-119 mL; 95% CI: 222 mL to 3mL) in post-menopausal women compared to with pre-menopausal women (Supplementary Table 5). No association was observed between menopausal status and age acceleration measures (Supplementary Table 6).

### Age stratified analysis in females

**Figure 3:**
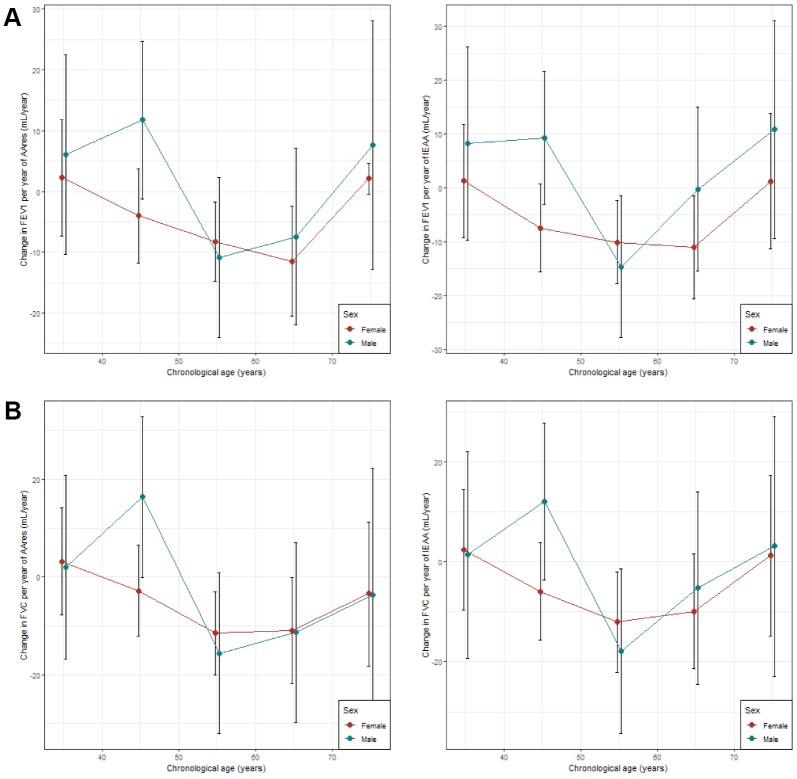
**Age stratified analyses comparing association between lung and age accelerations.** (**A**) Comparison between association between FEV_1_ and age accelerations (AAres and IEAA); (**B**) Comparison between association between FVC and age accelerations (AAres and IEAA). X-axis represents stratifications by 10 years intervals. Y-axis represents the estimates (changed in lung function per year of epigenetic age acceleration in mL/year) from the linear models with 95% confidence intervals.

Significant associations between lung function (FEV_1_ and FVC) and age acceleration (AA_res_ and IEAA) were observed both in male and female samples in the cross- sectional age stratified analysis. AA_res_ and IEAA were found to be significantly associated with FEV_1_ in age groups 50 – 60 and 60 – 70 in female samples (Supplementary Table 7A). The same trend was observed for FVC (Supplementary Table 7B). No significant association was observed for FEV_1_/FVC (Supplementary Table 7C). IEAA were found to be significantly associated with both FEV_1_ and FVC in age groups 50 – 60 in males. However, while consistent lung function decline (for FEV_1_ and FVC) per epigenetic year (for AA_res_ and IEAA) is found up to 70 years in females, the same trend is not observed in males (Figure 3, Supplementary Figure 2).

### Cross-sectional association between lung function and DNAmAge_grim_, AA_grim_, DNAmPAI1 and DNAmPAI1_adj_ at baseline and follow-up time point separately

Strong associations between lung function and DNAmAge_grim_ and AA_grim_ were observed respectively in the cross-sectional analyses for both baseline and follow-up time points (Table 6). The rate of changes in lung function declines are found stronger in follow-up years than that of the baseline for both DNAmAge_grim_ (FEV_1_: -12.72 mL/year to -30.14 mL/year; FVC: -7.56 mL/year to -29.42 mL/year; FEV_1_/FVC: -0.001 to -0.002) and AA_grim_ (FEV_1_: -11.96 mL/year to -29.35 mL/year; FVC: -6.64 mL/year to -28.48 mL/year; FEV_1_/FVC: -0.001 to -0.002), and the same trend is observed for AA_grim_. Similar strong associations between lung functions (FEV_1_ and FVC) and DNAmPAI1 and DNAmPAI1_adj_ were observed respectively in the cross-sectional analyses for both baseline and follow-up time points (Table 7). Though no association was found between FEV_1_/FVC, and DNAmPAI1 and DNAmPAI1_adj_ at baseline time point, a significant association was still observed at the follow-up time point. However, association between FEV_1_/FVC with DNAmAge_grim_, DNAmPAI1 and their associated age adjusted measures showed high level of heterogeneity at baseline time point (shown in heterogeneity p-values in Supplementary Figure 3).

**Table 6:** Cross-sectional meta-analysis results of association between DNAm GrimAge (DNAmAge_grim_) and its age-adjusted measure (AA_grim_) with lung function in SAPALDIA and ECRHS cohorts.

**Lung functions**	**Baseline**					**Follow-up**			
		**Estimate**	**Lower bound**	**Upper bound**	**P-value**	**Estimate**	**Lower bound**	**Upper bound**	**P-value**
FEV_1_	DNAmAge_grim_	-12.72	-17.91	-7.52	1.62 x 10^-06^*	-30.14	-37.05	-23.23	1.25 x 10^-17^*
	AA_grim_	-11.96	-17.17	-6.76	6.67 x 10^-06^*	-29.35	-36.32	-22.39	1.42 x 10^-16^*
									
FVC	DNAmAge_grim_	-7.56	-13.59	-1.53	0.01*	-29.42	-37.46	-21.39	7 x 10^-13^*
	AA_grim_	-6.64	-12.69	-0.59	0.03*	-28.48	-36.57	-20.39	5.25 x 10^-12^*
									
FEV_1_/FVC	DNAmAge_grim_	-0.001	-0.002	-0.0005	6.68 x 10^-04^*	-0.002	-0.003	-0.001	4.85 x 10^-06^*
	AA_grim_	-0.001	-0.002	-0.0005	7.25 x 10^-04^*	-0.002	-0.003	-0.001	7.06 x 10^-06^*

Here Estimate = changes in lung function per year of epigenetic age and age acceleration (mL/year for DNAmAge_grim;_ mL/year_AA_ for AA_grim_). Negative values denote that with every year of increase in epigenetic age acceleration, lung function decreases and vice-versa; Lower and upper = lower and upper ranges of 95% confidence interval of estimates; P-value = p-values from meta-analyses.

**Table 7:** Cross-sectional meta-analysis results of association between DNAm based plasminogen activation inhibitor 1 (DNAmPAI1) and its age adjusted (DNAmPAI1_adj_) levels with lung function in SAPALDIA and ECRHS cohorts.

**Lung functions**	**Baseline**					**Follow-up**			
		**Estimate**	**Lower bound**	**Upper bound**	**P-value**	**Estimate**	**Lower bound**	**Upper bound**	**P-value**
FEV_1_	DNAmPAI1	-0.020	-0.029	-0.011	8.85 x 10^-06^*	-0.032	-0.041	-0.022	3.63 x 10^-11^*
	DNAmPAI1_adj_	-0.019	-0.028	-0.010	2.57 x 10^-05^*	-0.031	-0.041	-0.022	9.67 x 10^-11^*
									
FVC	DNAmPAI1	-0.018	-0.028	-0.008	4.84 x 10^-04^*	-0.029	-0.039	-0.018	2.14 x 10^-07^*
	DNAmPAI1_adj_	-0.018	-0.029	-0.008	4.34 x 10^-04^*	-0.029	-0.040	-0.018	1.53 x 10^-07^*
									
FEV_1_/FVC	DNAmPAI1	-1 x 10^-6^	-2 x 10^-6^	0.00	0.14	-2x 10^-6^	-4 x 10^-6^	-1 x 10^-6^	0.002*
	DNAmPAI1_adj_	-1 x 10^-6^	-2 x 10^-6^	1 x 10^-6^	0.29	-2x 10^-6^	-3 x 10^-6^	-1 x 10^-6^	0.003*

Here, Estimate = difference in lung function (mL) per unit of DNAmPAI1 and DNAmPAI1_adj_. Negative values denote that with every year of increase in epigenetic age acceleration, lung function decreases and vice-versa; Lower and upper = lower and upper ranges of 95% confidence interval of estimates; P-value = p-values from meta-analyses.

### Association between lung function and DNAmAge_grim_, AA_grim_, DNAmPAI1 and DNAmPAI1_adj_ from repeated measures at baseline and follow-up time points combined

There was evidence for significant associations of lower FEV_1_ and FVC with DNAmAge_grim_, AA_grim,_, DNAmPAI1, and DNAmPAI1adj (Table 8). FEV_1_/FVC is only found significantly associated with DNAmAge_grim_ and AA_grim_. However, the associations between lung function and DNAmAge_grim_ and AA_grim_ exhibited high level of heterogeneity (shown in heterogeneity p-values in Supplementary Figure 4).

**Table 8:** Meta-analysis results of repeat cross-sectional association between DNAmAge_grim_, AA_grim_, DNAmPAI1, and DNAmPAI1_adj_ with lung function in SAPALDIA and ECRHS cohorts from two time points (baseline and follow-up).

**Lung functions**		**Estimate**	**Lower bound**	**Upper bound**	**P-value**
FEV_1_	DNAmAge_grim_	-12.91	-16.63	-9.19	1.03 x 10^-11^*
	AA_grim_	-12.28	-16.03	-8.53	1.37 x 10^-10^*
	DNAmPAI1	-0.013	-0.018	-0.008	1.71 x 10^-06^*
	DNAmPAI1_adj_	-0.0119	-0.0171	-0.0067	7.81 x 10^-06^*
FVC	DNAmAge_grim_	-12.29	-16.92	-7.66	2 x 10^-07^*
	AA_grim_	-11.12	-15.78	-6.45	2.98 x 10^-06^*
	DNAmPAI1	-0.020	-0.027	-0.013	1.14 x 10^-08^*
	DNAmPAI1_adj_	-0.019	-0.026	-0.012	3.47 x 10^-08^*
FEV_1_/FVC	DNAmAge_grim_	-0.0009	-0.002	-0.0003	0.002*
	AA_grim_	-0.0009	-0.002	-0.0003	0.002*
	DNAmPAI1	1 x 10^-7^	-8 x 10^-6^	9 x 10^-6^	0.99
	DNAmPAI1_adj_	9 x 10^-7^	-8 x 10^-6^	1 x 10^-5^	0.83

Here, Estimate = changes in lung function per year of epigenetic age and age acceleration (mL/year for DNAmAge_grim;_ mL/year_AA_ for AA_grim_) and per unit of PAI-1 for DNAmPAI1 and DNAmPAI1_adj_. Negative values denote that with every unit of increase in DNAmAge_grim_. AA_grim_, DNAmPAI1, and DNAmPAI1_adj_ lung function decreases and vice-versa; Lower and upper = lower and upper ranges of 95% confidence interval of estimates; P-value = p-values from meta-analyses.

## DISCUSSION

Using longitudinal data from two population-based cohorts we have examined the association of lung function with epigenetic aging and shown that lung function is associated with measures of epigenetic age acceleration, particularly in women and with increasing age**.** Lung function decline is found to be strongly associated with increase in DNA methylation-based lifespan predictors, plasma protein levels, and their related age adjusted measures.

This is one of the first studies to examine the association of age acceleration on lung function over more than one time point, and similar to the Mid-Lothian cohort showing marginal association of FEV_1_ with epigenetic age acceleration [30]. In the Mid-Lothian birth cohort study, participants were of older age (70 years at baseline, 76 years at follow-up) than the studies used here, whereas the present study investigates a wider and younger age range (37 to 61 years at baseline, 48 to 70 years at follow-up) and a follow-up time window of 8 to 11 years for SAPALDIA and ECRHS.

Our findings suggest that lung function is associated with age acceleration in women and particularly in women above age of 50 years. FEV_1_ was found to be declining at a rate of 9.5 mL per year of age acceleration using AA_res_ and 11.3 mL per year of age acceleration using IEAA. This same trend was observed for FVC. This observation was further supported by SAPALDIA baseline measures (which were in an older group of women) showing a greater effect of age acceleration on lung function decline than the ECRHS baseline.

Early menopause and post-menopausal status have previously been linked with lower lung function [24] and menopause has been shown to accelerate epigenetic aging of blood [21]. Mendelian randomization studies have supported a casual effect of menopause on IEAA [25]. Therefore, we postulated that one explanation for the stronger association of lung function with age acceleration in the older women could be hormonal changes. There was a marginally significant association (P-value < 0.1) in lung function decline in post-menopausal females compared with pre-menopausal females, and adjusting for menopausal status resulted associations between lung function and age acceleration became less strong. However, there were no significant differences between the two models. This suggests that the onset of menopause may only partially explain the stronger associations observed between age acceleration and lung function in older female subjects. We also observed no significant association between measures of epigenetic age acceleration and menopause in our study sample.

When the association from the repeated measures from two time points was assessed, a marginal association was found in female subjects, showing a 3.94 ml decline in FVC per year of epigenetic age acceleration (AA_res_). In contrast, while measuring the effect of age acceleration on lung function decline between baseline and follow-up, there were no significant associations, suggesting that decline in lung function is proportional to the overall degree of biological aging.

The most interesting results were achieved for DNAm based lifespan predictors, DNAmAge_grim_ and AA_grim_, which have been found strongly associated with lung function for both baseline and follow-up time points and combined. However, results for the combined repeated time points should be interpreted with caution due to the presence of indication of heterogeneity between two cohorts. DNAm based plasma protein levels, PAI-1 and age adjusted PAI-1, were also observed to be associated with lung function both cross-sectionally and in combined repeated measures. This association result is of particular interest as studies have shown elevated PAI-1 level to be associated with lung function decline [26, 27], which corroborates with our findings.

One limitation of this study is that we have used epigenetic age derived from blood rather than lung tissue to assess associations. However, epigenetic aging measured from blood has been found to be associated with a number of other non-blood related diseases and phenotypes such as lung cancer [28], metabolic syndrome [15], and developmental disorders [29]. Additionally, other physiological changes (such as hormonal changes) were not considered. Though we have used menopausal status in sensitivity analyses as a categorical variable, adding direct measures of sex hormone concentrations may provide more insight.

In conclusion, this study suggests that epigenetic age acceleration is significantly associated with lung function in women older than 50 years. We hypothesised that this could be due to menopause. However, we have observed that menopause has minimal effect and therefore there is possibility of other unknown physiological factors at older age in females mediating the epigenetic age acceleration effect on lung function. While, it is still unknown what exactly epigenetic aging from DNA methylation measures, this study suggests it can be utilised as one of the important factors to assess women’s lung health in old age. DNA methylation-based lifespan predictors, such as: DNAm GrimAge and plasma protein levels, are strongly associated with lung function and therefore this study suggests that these can be utilised as important factors to assess lung health in adults.

## MATERIALS AND METHODS

### Study population

Information from 1,496 participants taking part in either the Swiss study of Air Pollution and Lung and heart Disease in Adults (SAPALDIA) [30, 31] (N=987), or the European Community Respiratory Health Survey (ECRHS) [32] (N=509) were used in this investigation. Measures of lung function, relevant confounders and DNAm of the samples were taken at two time points (baseline and follow-up).

### DNA methylation

DNA for all cohorts was extracted from peripheral blood samples taken at two consecutive surveys 8 years apart in SAPALDIA and 11 years apart in ECRHS. Samples for testing were selected on the basis of having lung function complete and high quality of information on lung function and relevant confounders. Genome-wide DNA methylation was quantified using the Illumina Infinium HumanMethylation450 Beadchip for SAPALDIA samples and using the Illumina Infinium HumanEPIC Beadchip for ECRHS samples. Samples from two time points derived from the same subject were placed next to each other on the array to minimise batch effect. Sample and CpG marker quality control procedures for epigenetic data of both cohorts are described elsewhere [33].

### Measures of epigenetic aging

DNA methylation age (DNAmAge) was calculated using (a) the Horvath method [10] using 353 cytosine-phosphate-guanine sites (CpGs) common to the Illumina 450K and EPIC Methylation arrays, and (b) Hannum’s method using 71 CpGs [9]. Age acceleration residuals (AA_res_) were calculated from a linear regression model by regression of DNAmAge on chronological age. Further, AA_res_ measures were adjusted for blood cell counts to calculate Intrinsic Epigenetic Age Acceleration (IEAA) using the Horvath method and Extrinsic Epigenetic Age Acceleration (EEAA) using Hannum method, described in [13]. Age acceleration measures (IEAA and EEAA) were estimated using an online calculator (available from: https://dnamage.genetics.ucla.edu/submit). DNAm based GrimAge and its associated age acceleration measures (DNAmAge_grim_ and AA_grim_) and DNAm-based estimators of plasma proteins and its age adjusted level (DNAmPAI1 and DNAmPAI1_adj_) were calculated using the new online calculator (available from: https://dnamage.genetics.ucla.edu/new)

### Lung function measures

Two objective measures of lung function, forced expiratory volume in one second (FEV_1_) and forced vital capacity (FVC), and their ratio (FEV_1_/FVC) were examined. They were measured by trained personnel according to the ATS/ERS recommendations [34]. Lung function measures for SAPALDIA was obtained from 2001 and 2010 measurements with correction for change in spirometers (SensorMedics to Easy One : ndd Medical Technologies, Zurich, Switzerland) [35]. For ECRHS, different spirometers were used in each centre (Biomedin in the UK, Sensor Medics in Norway and Jaeger Pneumolab in Germany) at baseline and the same spirometer (Easy One: ndd Medical Technologies, Zurich, Switzerland) was used in all centres at follow-up.

### Covariates

Analyses were adjusted for age, sex, height (cm), body mass index (kg/m^2^), self-reported history of lifetime asthma, level of completed education as a proxy for socio-economic status, and smoking status (never, former, current) for both time points. The study centres were also considered as covariates as the samples were distributed over multiple geographical areas.

### Statistical analysis

To assess the variability between chronological age and DNAmAge, correlation and Median Absolute Deviation (MAD) were determined. To assess the association of lung function with age acceleration (AA) cross-sectionally, linear regression was used for each of the two time points (approximately 10 years apart) with lung function as the outcome and AA as predictor while adjusting for all covariates (Model 1). Secondly, a linear mixed model was used to assess the association of lung function with age acceleration by combining the available data at both time points for each individual, adjusting for all covariates from both time points (Model 2). This model incorporates sample and time point differences by introducing random intercepts for individuals and time points. In the third model, the association of lung function change (mL/year) with change in DNAmAge from baseline and follow-up (calculated from the difference between DNAmAge between two time points) was assessed (Model 3). The rate of lung function change was defined by $\frac{Lung\;function_{follow-up}\;-\;Lung\;function_{base}}{Age_{follow-up}-Age_{base}}$. For Model 3 average BMI, average height, educational status at any time point, transition in asthma status, change in smoking status and centres were used as covariates. Samples with discrepancies in educational status and centres were removed. As epigenetic age acceleration has previously been found to be strongly associated with sex [36] (Supplementary Figure 1), and thus all models were *a priori* stratified by sex. In addition, the association between lung function and DNAmAge_grim_, AA_grim_, DNAmPAI1, and DNAmPAI1_adj_ were assessed individually following Models 1 and 2. In this case, the associations were not stratified by sex. All models were performed in each cohort separately and effect estimates were meta-analysed using a fixed-effect model weighted by the inverse of the variance, using the R package “metafor” [37].

Associations of AA, except AA_grim_, with lung function were further explored using an age stratified analysis (by 10 years: 30 – 40 years, 40 – 50 years, 50 – 60 years, 60 – 70 years, and 70 – 80 years) using a linear mixed effects model.

Further analyses were undertaken on female subjects at follow-up using menopausal status (pre-, peri-, and post-menopausal) to identify the effect of menopause on lung function and age accelerations using linear models adjusted for above mentioned covariates. The classifications of menopausal status for individual cohort have been described elsewhere [24, 38]. All statistical analyses were performed with R v3.3.2 [39].

## Supplementary Material

Supplementary Materials

Supplementary Figures

Supplementary Tables

Supplementary Table 1

Supplementary Table 7
