# Supplementary material for: Association of adult lung function with accelerated biological aging
Source: Aging (Albany NY). 2020 Jan 11;12(1):518–42. doi: 10.18632/aging.102639 (PMC6977706; doi:10.18632/aging.102639)
Supplement: Supplementary Materials [file aging-12-102639-s003..pdf]

SUPPLEMENTARY MATERIALS

COHORT SPECIFIC FUNDING AND ACKNOWLEDGEMENT

SAPALDIA - Swiss study on air pollution heart and lung disease in adults

ACKNOWLEDGMENTS

The study could not have been done without the help of the study participants, technical and administrative support and the medical teams and field workers at the local study sites.

Study directorate: NM Probst-Hensch (PI; e/g); T Rochat (p), C Schindler (s), N Künzli (e/exp), JM Gaspoz (c)

Scientific team: JC Barthélémy (c), W Berger (g), R Bettschart (p), A Bircher (a), C Brombach (n), PO Bridevaux (p), L Burdet (p), Felber Dietrich D (e), M Frey (p), U Frey (pd), MW Gerbase (p), D Gold (e), E de Groot (c), W Karrer (p), F Kronenberg (g), B Martin (pa), A Mehta (e), D Miedinger (o), M Pons (p), F Roche (c), T Rothe (p), P Schmid-Grendelmeyer (a), D Stolz (p), A Schmidt-Trucksäss (pa), J Schwartz (e), A Turk (p), A von Eckardstein (cc), E Zemp Stutz (e).

Scientific team at coordinating centers: M Adam (e), I Aguilera (exp), S Brunner (s), D Carballo (c), S Caviezel (pa), I Curjuric (e), A Di Pascale (s), J Dratva (e), R Ducret (s), E Dupuis Lozeron (s), M Eeftens (exp), I Eze (e), E Fischer (g), M Foraster (e), M Germond (s), L Grize (s), S Hansen (e), A Hensel (s), M Imboden (g), A Ineichen (exp), A Jeong (g), D Keidel (s), A Kumar (g), N Maire (s), A Mehta (e), R Meier (exp), E Schaffner (s), T Schikowski (e), M Tsai (exp) (a) allergology, (c) cardiology, (cc) clinical chemistry, (e) epidemiology, (exp) exposure, (g) genetic and molecular biology, (m) meteorology, (n) nutrition, (o) occupational health, (p) pneumology, (pa) physical activity, (pd) pediatrics, (s) statistics

FUNDING

The Swiss National Science Foundation (grants no 33CS30-148470/1&2, 33CSCO-134276/1, 33CSCO108796, 324730_135673, 3247BO-104283, 3247BO-104288, 3247BO-104284, 3247-065896, 3100-059302, 3200-052720, 3200-042532, 4026-028099, PMPDP3_129021/1, PMPDP3_141671/1), the Federal Office for the Environment, the Federal Office of Public Health, the Federal Office of Roads and Transport, the canton's government of Aargau, Basel-Stadt, Basel-Land, Geneva, Luzern, Ticino, Valais, and Zürich, the Swiss Lung League, the canton's Lung League of Basel Stadt/ Basel Landschaft, Geneva, Ticino, Valais, Graubünden and Zurich, Stiftung ehemals Bündner Heilstätten, SUVA, Freiwillige Akademische Gesellschaft, UBS Wealth Foundation, Talecris Biotherapeutics GmbH, Abbott Diagnostics, European Commission 018996 (GABRIEL), Wellcome Trust WT 084703MA, Exposomics EC FP7 grant(Grant agreement No: 308610).

ECRHS - european community respiratory health survey

ACKNOWLEDGMENTS

The authors would like to thank the participants, field workers and researchers who have participated in the ECRHS study for their time and cooperation.

FUNDING

This work was supported by a contract from the European Commission (018996), Fondo de Investigación Sanitaria (91/0016-060-05/E, 92/0319, 93/0393, 97/0035-01, 99/0034-01 and 99/0034-02), Hospital General de Albacete, Hospital General Ramón Jiménez, Consejería de Sanidad del Principado de Asturias, CIRIT (1997SGR 00079, 1999SGR 00241), and Servicio Andaluz de Salud, SEPAR, Public Health Service (R01 HL62633-01), RCESP (C03/09), Red RESPIRA (C03/011), Basque Health Department, Swiss National Science Foundation, Swiss Federal Office for Education and Science, Swiss National Accident Insurance Fund (SUVA), GSF-National Research Centre for Environment and Health, Deutsche Forschungsgemeinschaft (DFG) (FR 1526/1-1, MA 711/4-1), Programme Hospitalier de Recherche Clinique-DRC de Grenoble 2000 no. 2610, Ministry of Health, Direction de la Recherche Clinique, Ministere de l'Emploi et de la Solidarite, Direction Generale de la Sante, CHU de Grenoble, Comite des Maladies Respiratoires de l'Isere. UCB-Pharma (France), Aventis (France), Glaxo France. Estonian Science Foundation, and Asthma UK (formerly known as National Asthma Campaign UK).
