# Supplementary material for: Association of adult lung function with accelerated biological aging
Source: Aging (Albany NY). 2020 Jan 11;12(1):518–42. doi: 10.18632/aging.102639 (PMC6977706; doi:10.18632/aging.102639)
Supplement: Supplementary Figures [file aging-12-102639-s005..pdf]

## SUPPLEMENTARY FIGURES

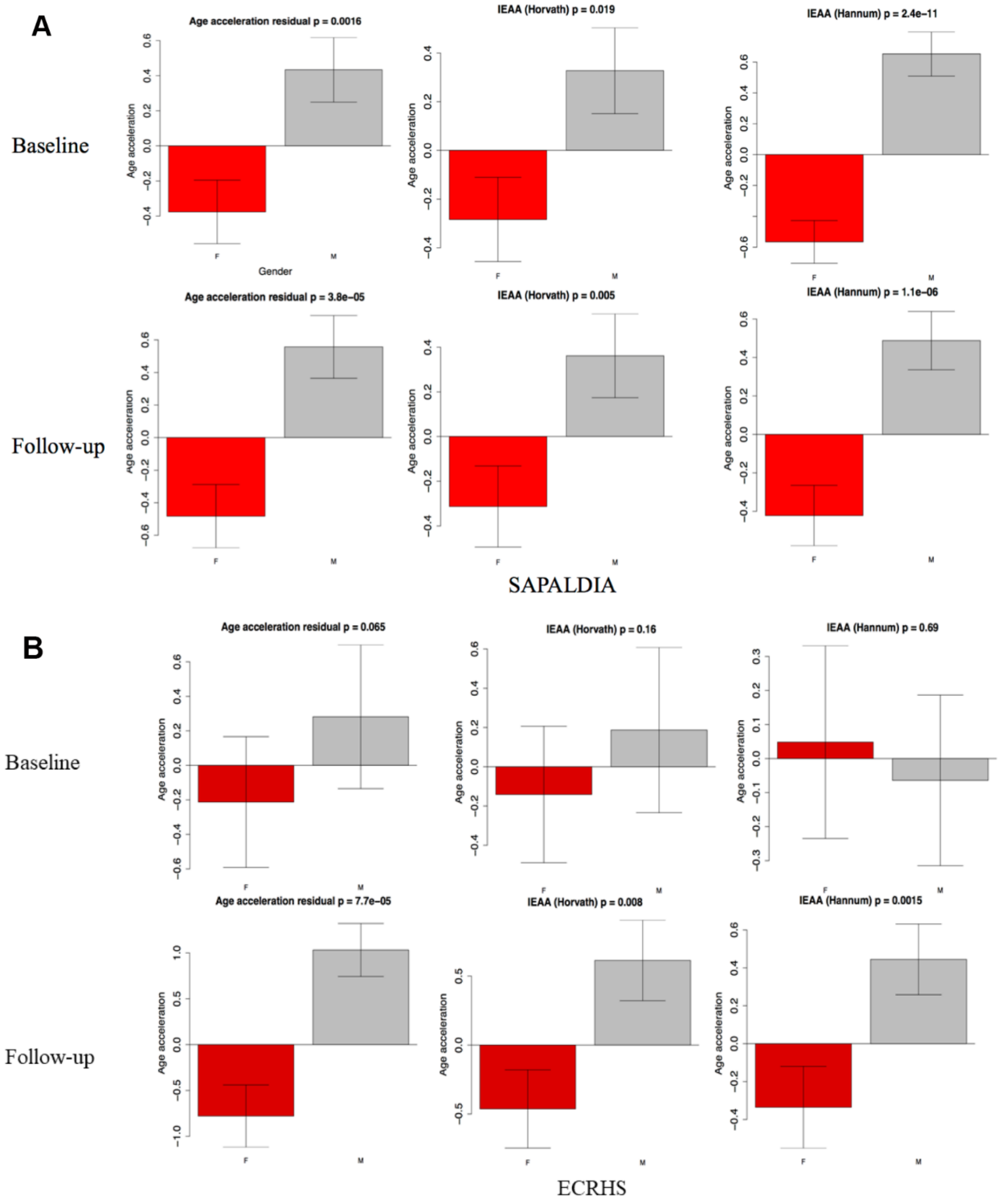


**Supplementary Figure 1. Sex-specific effect on epigenetic age acceleration.**

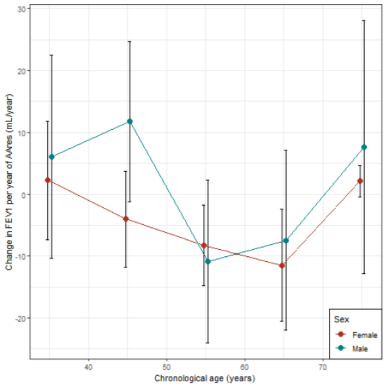


**A** Changes in $FEV_1$ per year of $AA_{res}$

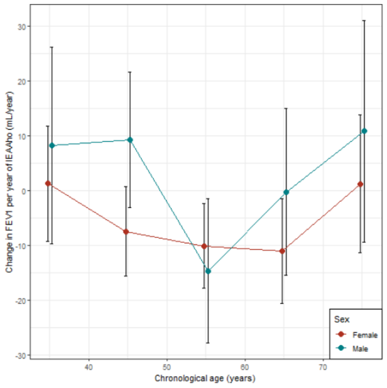


**B** Changes in $FEV_1$ per year of IEAA

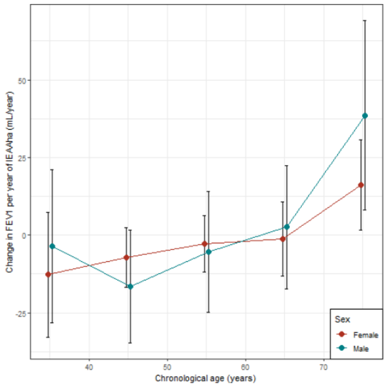


**C** Changes in $FEV_1$ per year of EEAA

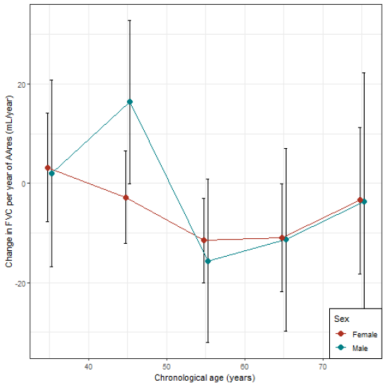


**D** Changes in FVC per year of $AA_{res}$

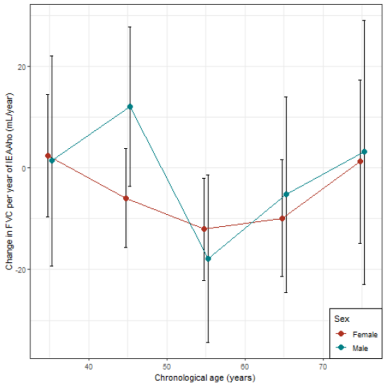


**E** Changes in FVC per year of IEAA

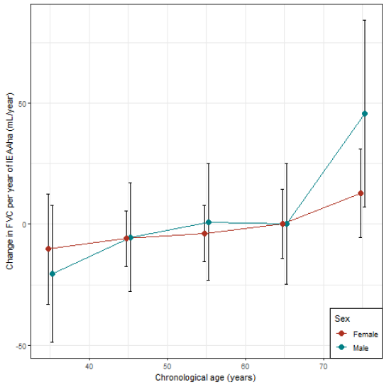


**F** Changes in FVC per year of EEAA

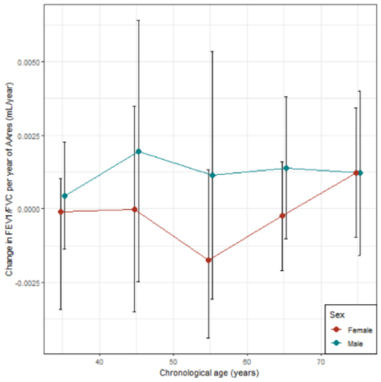


**G** Changes in $FEV_1$/FVC per year of $AA_{res}$

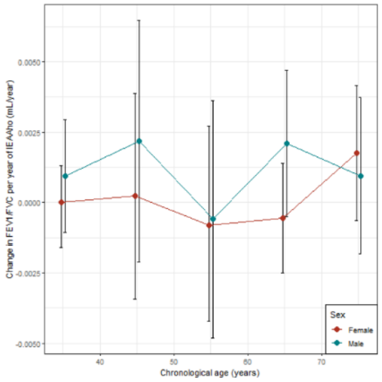


**H** Changes in $FEV_1$/FVC per year of IEAA

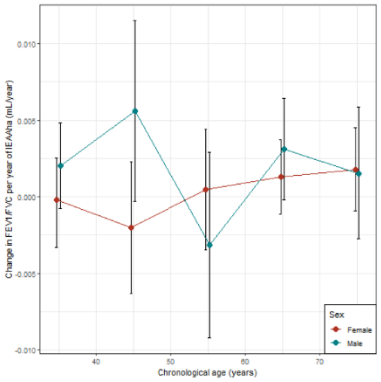


**I** Changes in $FEV_1$/FVC per year of EEAA

**Supplementary Figure 2. Age stratified analyses comparing association between lung function and epigenetic age accelerations.** X-axis represents stratifications by 10 years intervals. Y-axis represents the estimates (changed in lung function per year of epigenetic age acceleration in mLs/year) from the linear models with 95% confidence intervals.

**A** DNAmAge$_{grim}$

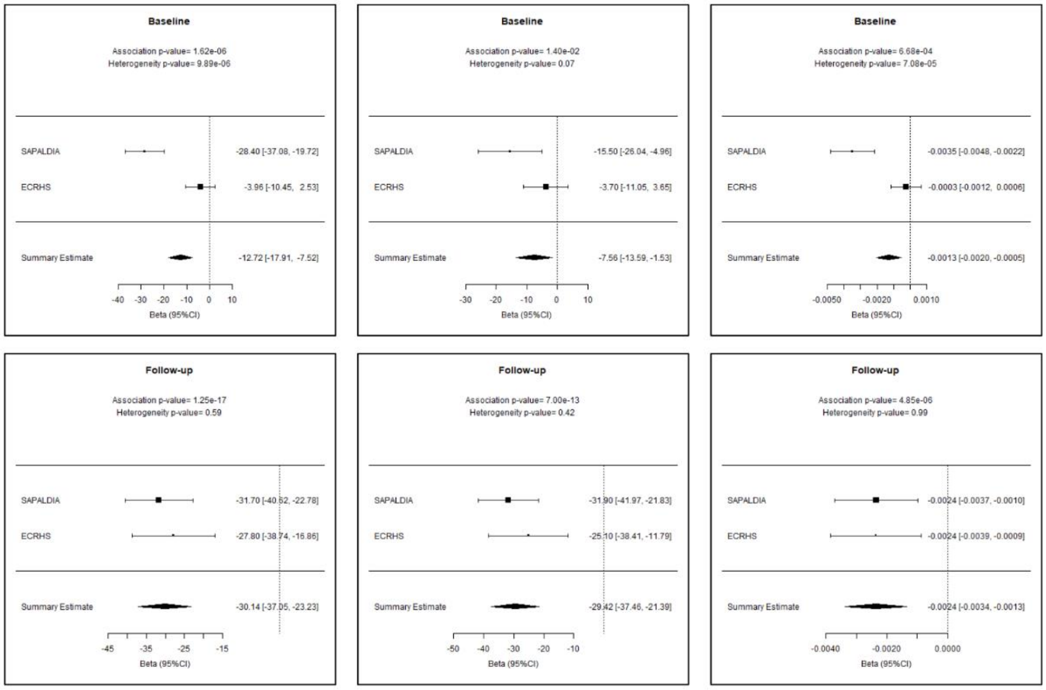


FEV$_1$ **FVC** FEV$_1$/**FVC**

**B** AA$_{grim}$

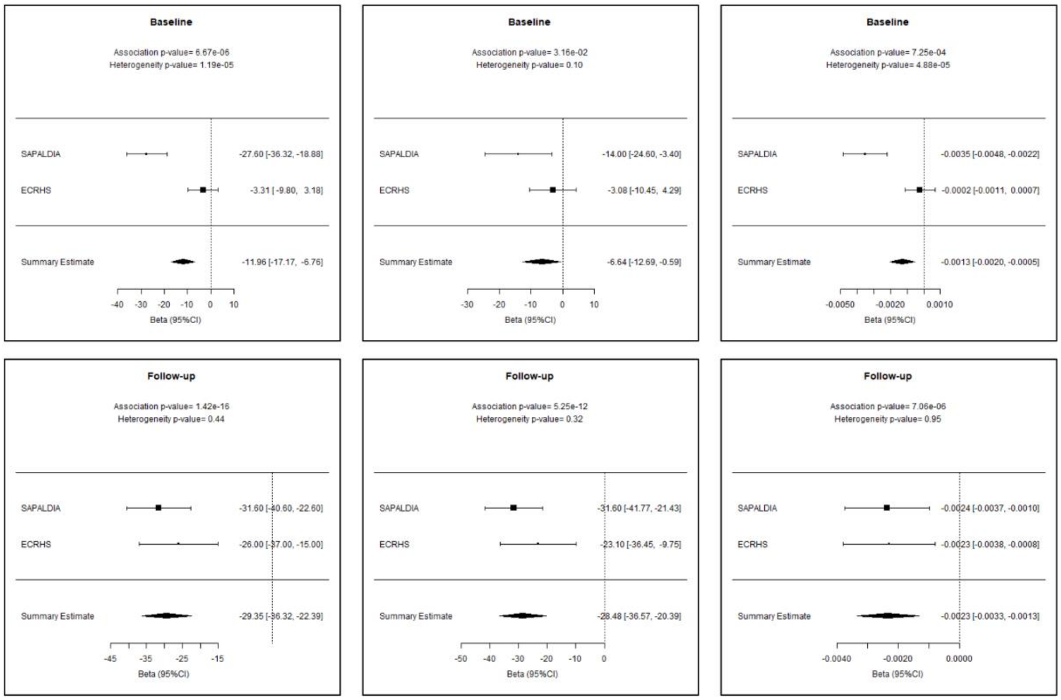


FEV$_1$ **FVC** FEV$_1$/**FVC**

**C** DNAmPAI1

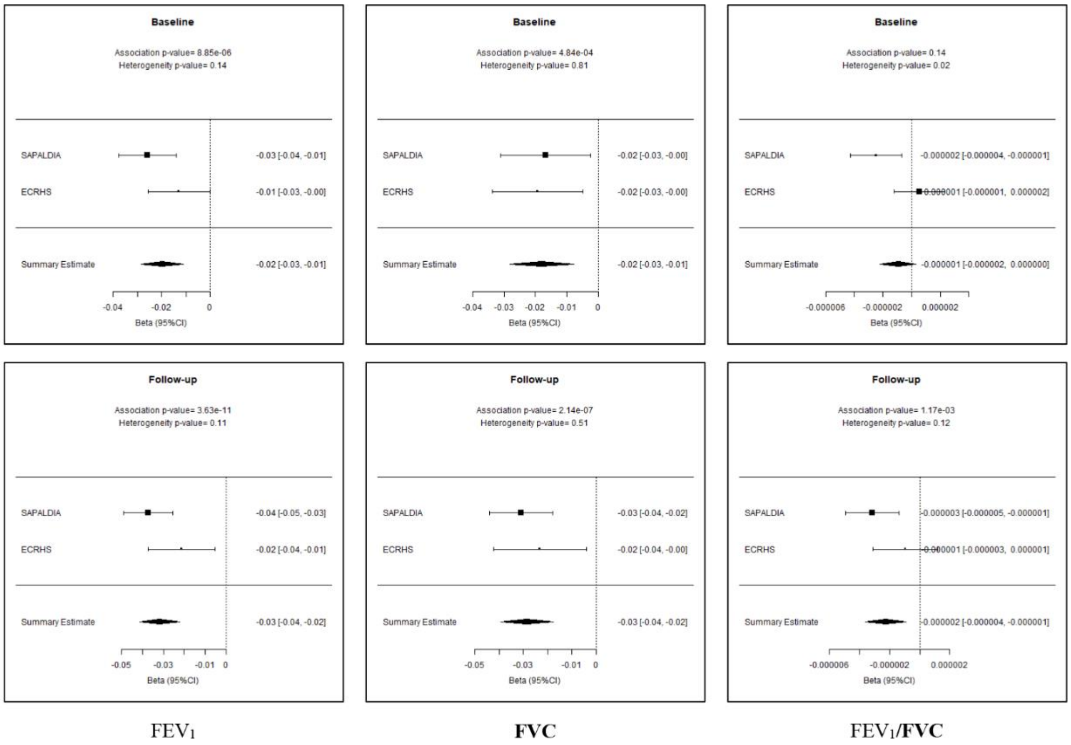


**D** DNAmPAI1$_{adj}$

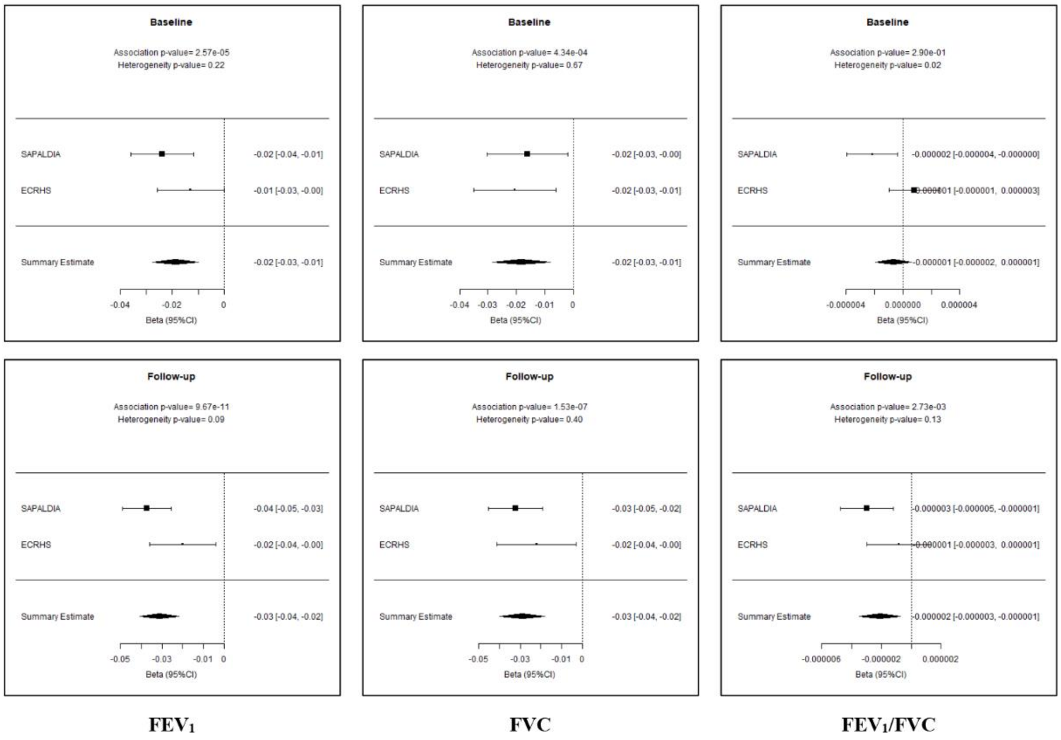


**Supplementary Figure 3. Cross-sectional meta-analysis results of association between DNAm GrimAge (DNAmGrim), Age-adjusted measure of DNAm GrimAge ($AA_{grim}$), DNAm plasminogen activation inhibitor 1 (DNAmPAI1) and Age adjusted DNAm PAI-1 (DNAmPAI1$_{adj}$) with lung function ($FEV_1$, FVC, and $FEV_1$/FVC) in SAPALDIA and ECRHS cohorts.** The estimates have been measured with 95% confidence interval.

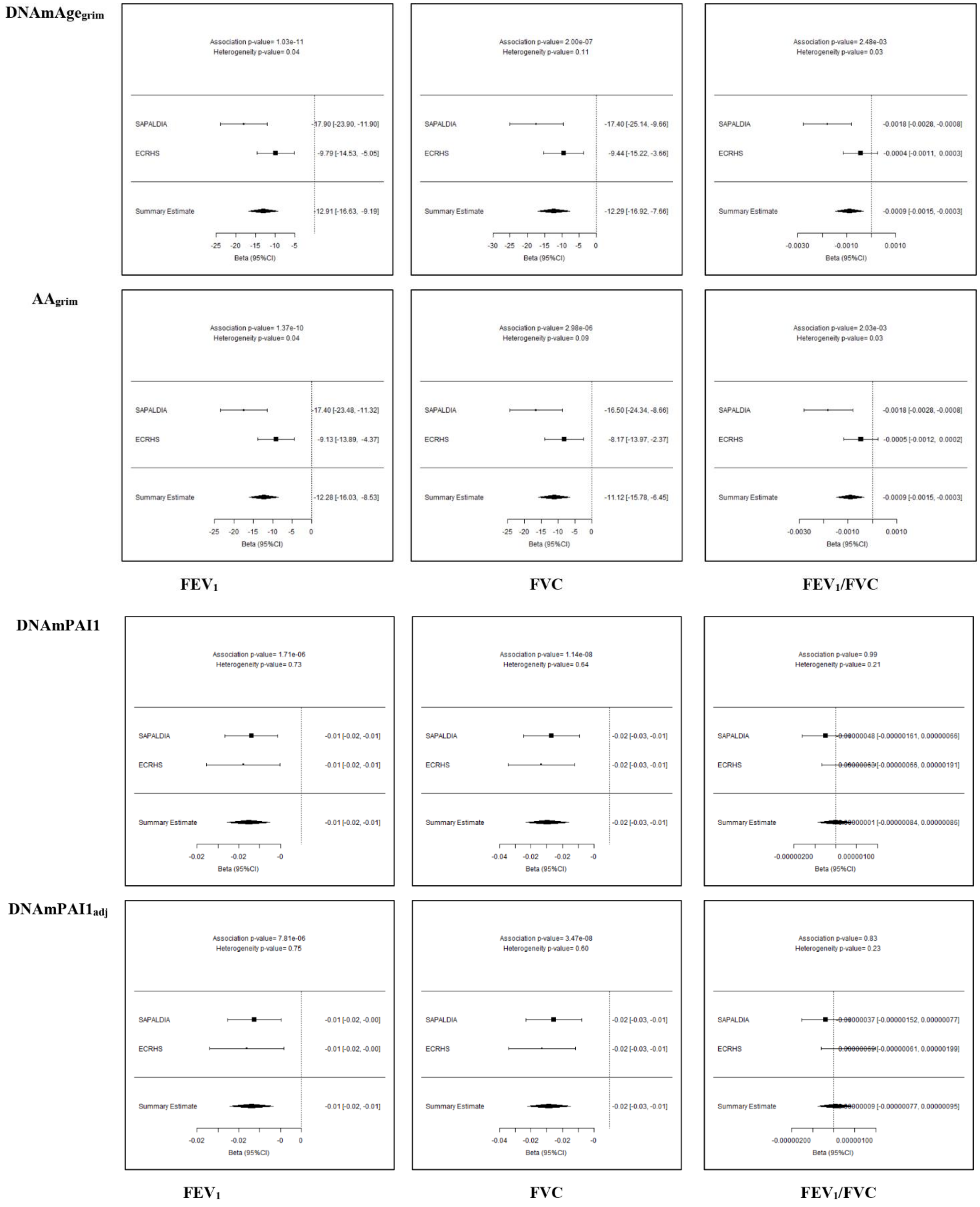


**Supplementary Figure 4. Linear mixed model meta-analysis association of lung function ($FEV_1$, FVC, and $FEV_1$/FVC) with DNAm GrimAge (DNAmGrim), Age-adjusted measure of DNAm GrimAge ($AA_{grim}$), DNAm plasminogen activation inhibitor 1 (DNAmPAI1) and Age adjusted DNAm PAI-1 ($DNAmPAI1_{adj}$) two time points (baseline and follow-up).** The estimates have been measured with 95% confidence interval.
