# Supplementary material for: Association of adult lung function with accelerated biological aging
Source: Aging (Albany NY). 2020 Jan 11;12(1):518–42. doi: 10.18632/aging.102639 (PMC6977706; doi:10.18632/aging.102639)
Supplement: Supplementary Tables [file aging-12-102639-s004..pdf]

SUPPLEMENTARY TABLES

Please browse Full Text version to see the data of Supplementary Tables 1

Supplementary Table 1. Cross-sectional meta-analysis results of association between age acceleration and lung function of males and females in SAPALDIA and ECRHS. SAP = SAPALIDA, ECR = ECRHS. Lower and upper is the lower and upper ranges of 95% confidence interval of estimates.

Supplementary Table 2. Longitudinal meta-analysis results of association between age acceleration and lung function of males and females in SAPALDIA and ECRHS. SAP = SAPALIDA, ECR = ECRHS.

A. Male

Age acceleration	Lung function	Cohort	Estimate	Lower	Upper	P-value	Meta p-value
AA_{res}	FEV_1	SAP	0.75	-6.33	7.84	0.83	0.61
		ECR	1.53	-4.68	7.67	0.62	
	FVC	SAP	-2.35	-11.7	7.05	0.62	0.82
		ECR	0.49	-7.54	8.44	0.90	
	FEV_1/FVC	SAP	0.0002	-0.0009	0.001	0.72	0.54
		ECR	0.0002	-0.0007	0.001	0.62	
IEAA	FEV_1	SAP	2.86	-4.37	10.1	0.44	0.15
		ECR	3.46	-2.34	9.16	0.23	
	FVC	SAP	-1.01	-10.6	8.64	0.84	0.96
		ECR	0.85	-6.73	8.32	0.82	
	FEV_1/FVC	SAP	0.0004	-0.0008	0.002	0.55	0.25
		ECR	0.0004	-0.0004	0.001	0.33	
EEAA	FEV_1	SAP	-0.70	-9.60	8.19	0.88	0.13
		ECR	−10.10	−19.74	-0.46	0.04	
	FVC	SAP	−3.55	−15.43	8.33	0.56	0.4
		ECR	−3.84	-16.36	8.68	0.55	
	FEV_1/FVC	SAP	-0.0002	-0.001	0.0009	0.43	0.43
		ECR	-0.0003	-0.001	0.0006	0.05	

B. Female

Age acceleration	Lung function	Cohort	Estimate	Lower	Upper	P-value	Meta- p-value
AA_{res}	FEV_1	SAP	-4.12	-8.35	0.108	0.056	0.23
		ECR	-0.09	-3.34	3.13	0.96	
	FVC	SAP	-3.94	-9.78	1.88	0.18	0.06
		ECR	-2.84	-7.06	1.36	0.19	
	FEV_1/FVC	SAP	-0.0005	-0.001	0.0004	0.29	1.0
		ECR	0.0002	-0.0004	0.0008	0.48	
IEAA	FEV_1	SAP	-3.66	-8.1	0.77	0.11	0.34
		ECR	0.22	-3.57	3.98	0.91	
	FVC	SAP	-2.8	-8.93	3.31	0.37	0.13
		ECR	-2.99	-7.89	1.88	0.23	
	FEV_1/FVC	SAP	-0.0005	-0.001	0.0005	0.36	0.98
		ECR	0.0003	-0.0004	0.0009	0.48	
EEAA	FEV_1	SAP	−3.21	−8.66	2.24	0.25	0.05
		ECR	−3.88	−8.74	0.98	0.12	
	FVC	SAP	−2.43	−9.90	5.04	0.52	0.13
		ECR	−4.60	−10.91	1.71	0.15	
	FEV_1/FVC	SAP	-0.0002	-0.001	0.0006	0.29	0.28
		ECR	-0.0005	-0.001	0.0002	0.58	

Lower and upper is the lower and upper ranges of 95% confidence interval of estimates.

Supplementary Table 3. Longitudinal meta-analysis results of association between age acceleration and rate of lung function changes over baseline and follow-up of males and females in SAPALDIA and ECRHS.

A. Male

Age acceleration	Lung function	Cohort	Estimate	Lower	Upper	P-value	Meta p-value
AA_{res}	FEV_1	SAP	0.28	-0.80	1.36	0.61	0.22
		ECR	0.39	-0.29	1.06	0.26	
	FVC	SAP	0.54	-1.19	2.27	0.54	0.17
		ECR	0.57	-0.36	1.5	0.23	
	FEV_1/FVC	SAP	$-6.2x10^{-05}$	-0.0002	0.0001	0.50	0.92
		ECR	$1.4x10^{-05}$	$-9.1x10^{-05}$	0.0001	0.79	
IEAA	FEV_1	SAP	0.55	-0.52	1.63	0.31	0.75
		ECR	0.51	-0.12	1.13	0.11	
	FVC	SAP	0.44	-1.29	2.17	0.62	0.11
		ECR	0.67	-0.19	1.54	0.13	
	FEV_1/FVC	SAP	$-1.2x10^{-05}$	-0.0002	0.0002	0.90	0.06
		ECR	$2.1x10^{-05}$	$-7.7 x10^{-05}$	0.0001	0.67	
EEAA	FEV_1	SAP	0.20	-1.13	1.54	0.76	0.51
		ECR	0.51	-0.12	1.13	0.11	
	FVC	SAP	0.21	-1.94	2.35	0.85	0.57
		ECR	0.67	-0.19	1.54	0.13	
	FEV_1/FVC	SAP	$5.8 x10^{-05}$	-0.0002	0.0003	0.60	0.98
		ECR	$2.1 x10^{-05}$	$-7.7 x10^{-05}$	0.0001	0.67	

B. Female

Age acceleration	Lung function	Cohort	Estimate	Lower	Upper	P-value	Meta p-value
AA_{res}	FEV_1	SAP	-0.23	-0.84	0.39	0.47	0.38
		ECR	0.22	-0.10	0.54	0.17	
	FVC	SAP	0.22	-0.69	1.13	0.64	0.98
		ECR	-0.05	-0.47	0.36	0.81	
	FEV_1/FVC	SAP	-0.0001	-0.0003	$2.97 x10^{-05}$	0.12	0.17
		ECR	$6.2 x10^{-05}$	$4.3 x10^{-05}$	0.0001	0.04	
IEAA	FEV_1	SAP	-0.18	-0.82	0.46	0.58	0.29
		ECR	0.30	-0.08	0.67	0.12	
	FVC	SAP	0.34	-0.60	1.29	0.48	0.81
		ECR	-0.02	-0.51	0.47	0.92	
	FEV_1/FVC	SAP	-0.0001	-0.0003	$4.5 x10^{-05}$	0.17	0.16
		ECR	$7.5 x10^{-05}$	$6.7 x10^{-05}$	0.0001	0.03	
EEAA	FEV_1	SAP	-0.12	-0.91	0.68	0.77	0.59
		ECR	0.30	-0.08	0.67	0.12	
	FVC	SAP	0.52	-0.66	1.69	0.39	0.62
		ECR	-0.02	-0.51	0.47	0.92	
	FEV_1/FVC	SAP	-0.0002	-0.0004	$-3.3 x10^{-06}$	0.05	0.98
		ECR	$7.5 x10^{-05}$	$6.7 x10^{-06}$	0.0001	0.032	

Supplementary Table 4. Comparison between two meta-analyses models (with and without menopausal status). The difference (z-score) between the estimates from two models is calculated by Wald type test by the following equation:

$$z = \frac{\mu_1 - \mu_2}{\sqrt{SE[\mu_1]^2 + SE[\mu_2]^2}}$$

Here, μ_1 μ_2 are estimates and $SE[\mu_1]$, $SE[\mu_2]$ are corresponding standard errors of two meta-analyses.

		z -score	P-value
AA_{res}	FEV_1	-0.042	0.966
	FVC	-0.009	0.993
	FEV_1/FVC	0.779	0.28
IEAA	FEV_1	-0.087	0.931
	FVC	-0.044	0.965
	FEV_1/FVC	0.416	0.677
EEAA	FEV_1	-0.105	0.917
	FVC	-0.09	0.928
	FEV_1/FVC	0.095	0.924

Supplementary Table 5. Association between menopausal status and lung function.

	Lung function	Estimate	Lower	Upper	P-value
Pre- vs. Peri-menopause	FEV_1	-6	-130	117	0.919
	FVC	11	-140	163	0.882
	FEV_1/FVC	-0.002	-0.019	0.016	0.845
Pre- vs. Post-menopause	FEV_1	-109	-222	3	0.057
	FVC	-83	-212	46	0.209
	FEV_1/FVC	-0.009	-0.027	0.01	0.353
Peri- vs. Post-menopause	FEV_1	-62	-153	29	0.18
	FVC	-27	-130	76	0.607
	FEV_1/FVC	-0.011	-0.026	0.005	0.175

The units for estimates of FEV_1 and FVC are in mL. Lower and upper is the lower and upper ranges of 95% confidence interval of estimates.

Supplementary Table 6. Association between menopausal status and age acceleration.

		Beta	Lower	Upper	P-value
Pre- vs. Peri-menopause	AA_{res}	-0.135	-1.27	0.997	0.814
	IEAA	-0.397	-1.5	0.704	0.478
	EEAA	0.248	-0.617	1.11	0.573
Pre- vs. Post-menopause	AA_{res}	-0.129	-0.971	0.714	0.764
	IEAA	-0.221	-1.02	0.575	0.585
	EEAA	-0.0345	-0.69	0.62	0.918
Peri- vs. Post-menopause	AA_{res}	0.007	-0.948	0.961	0.989
	IEAA	0.176	-0.738	1.09	0.705
	EEAA	-0.282	-1.04	0.479	0.467

The units for estimate of age accelerations are in years. Lower and upper is the lower and upper ranges of 95% confidence interval of estimates.

Please browse Full Text version to see the data of Supplementary Tables 7

Supplementary Table 7. Age stratified cross-sectional analysis results of association between age acceleration and lung function of females in SAPALDIA and ECRHS. Lower and upper is the lower and upper ranges of 95% confidence interval of estimates.
