# Supplementary material for: Association of adult lung function with accelerated biological aging
Source: Aging (Albany NY). 2020 Jan 11;12(1):518–42. doi: 10.18632/aging.102639 (PMC6977706; doi:10.18632/aging.102639)
Supplement: Supplementary Table 1 [file aging-12-102639-s002..docx]

**Supplementary Table 1.** **Cross-sectional meta-analysis results of association between age acceleration and lung function of males and females in SAPALDIA and ECRHS.**

a. FEV_1_

| Sex | Time point | Age acceleration | Cohort | Estimate | Lower | Upper | P-value | Meta  p-value |
| --- | --- | --- | --- | --- | --- | --- | --- | --- |
| Male | Baseline | AA_res_ | SAP | 8.45 | -3.57 | 20.5 | 0.17 | 0.75 |
|  |  |  | ECR | -8.67 | -19.2 | 1.86 | 0.11 |  |
|  |  | IEAA | SAP | 9.51 | -3.06 | 22.1 | 0.14 | 0.55 |
|  |  |  | ECR | -10.9 | -21.5 | -0.314 | 0.04 |  |
|  |  | EEAA | SAP | -2.08 | -13 | 0.009 | 0.70 | 0.81 |
|  |  |  | ECR | 0.161 | -0.01 | 0.01 | 0.98 |  |
|  | Follow-up | AA_res_ | SAP | -4.37 | -16 | 7.25 | 0.46 | 0.24 |
|  |  |  | ECR | -7.03 | -21.8 | 7.7 | 0.35 |  |
|  |  | IEAA | SAP | -2.16 | -14.1 | 9.81 | 0.72 | 0.27 |
|  |  |  | ECR | -9.52 | -24 | 4.97 | 0.20 |  |
|  |  | EEAA | SAP | -10.7 | -21.3 | -0.13 | 0.05 | 0.13 |
|  |  |  | ECR | 4.53 | -12.2 | 21.3 | 0.59 |  |
|  |  |  |  |  |  |  |  |  |
| Female | Baseline | AA_res_ | SAP | -5.28 | -13.4 | 2.87 | 0.20 | 0.14 |
|  |  |  | ECR | -2.94 | -9.85 | 3.96 | 0.40 |  |
|  |  | IEAA | SAP | -5.53 | -14.1 | 3.02 | 0.20 | 0.08^†^ |
|  |  |  | ECR | -4.6 | -12.1 | 2.86 | 0.23 |  |
|  |  | EEAA | SAP | -2.91 | -10.1 | 4.24 | 0.42 | 0.09 |
|  |  |  | ECR | -5.78 | -12.9 | 1.37 | 0.11 |  |
|  | Follow-up | AA_res_ | SAP | -8.67 | -16.1 | -1.29 | 0.02 | 4 x 10^-04^* |
|  |  |  | ECR | -10.4 | -17.9 | -2.89 | 0.007 |  |
|  |  | IEAA | SAP | -9.88 | -17.8 | -1.96 | 0.01 | 2 x 10^-04^* |
|  |  |  | ECR | -13.1 | -22 | -4.14 | 0.004 |  |
|  |  | EEAA | SAP | -4.86 | -10.9 | 1.22 | 0.12 | 0.05 |
|  |  |  | ECR | -5.58 | -15.0 | 3.81 | 0.24 |  |
|  |  |  |  |  |  |  |  |  |

b. FVC

| Sex | Time point | Age acceleration | Cohort | Estimate | Lower | Upper | P-value | Meta  p-value |
| --- | --- | --- | --- | --- | --- | --- | --- | --- |
| Male | Baseline | AA_res_ | SAP | 2.04 | -11.6 | 15.7 | 0.77 | 0.36 |
|  |  |  | ECR | -9.23 | -21.4 | 2.97 | 0.14 |  |
|  |  | IEAA | SAP | 3.49 | -10.8 | 17.8 | 0.63 | 0.29 |
|  |  |  | ECR | -11.3 | -23.6 | 0.969 | 0.07 |  |
|  |  | EEAA | SAP | -3.31 | -15.4 | 8.76 | 0.59 | 0.87 |
|  |  |  | ECR | 4.74 | -7.46 | 16.9 | 0.45 |  |
|  | Follow-up | AA_res_ | SAP | -10.6 | -22.9 | 1.81 | 0.09 | 0.04* |
|  |  |  | ECR | -11.3 | -29.1 | 6.47 | 0.21 |  |
|  |  | IEAA | SAP | -7.62 | -20.4 | 5.15 | 0.24 | 0.08 |
|  |  |  | ECR | -12.4 | -29.9 | 5.15 | 0.17 |  |
|  |  | EEAA | SAP | -11.9 | -23.2 | -0.621 | 0.04 | 0.11 |
|  |  |  | ECR | 4.25 | -16 | 24.5 | 0.68 |  |
|  |  |  |  |  |  |  |  |  |
| Female | Baseline | AA_res_ | SAP | -7.81 | -18 | 2.39 | 0.13 | 0.13 |
|  |  |  | ECR | -2.88 | -10.4 | 4.63 | 0.45 |  |
|  |  | IEAA | SAP | -6.33 | -17 | 4.36 | 0.25 | 0.11 |
|  |  |  | ECR | -4.57 | -12.7 | 3.55 | 0.27 |  |
|  |  | EEAA | SAP | -5.44 | -14.3 | 3.47 | 0.23 | 0.12 |
|  |  |  | ECR | -3.96 | 11.8 | 3.85 | 0.26 |  |
|  | Follow-up | AA_res_ | SAP | -8.97 | -17.5 | -0.475 | 0.04 | 0.003* |
|  |  |  | ECR | -9.68 | -18.5 | -0.831 | 0.03 |  |
|  |  | IEAA | SAP | -10.4 | -19.5 | -1.26 | 0.03 | 0.003* |
|  |  |  | ECR | -10.6 | -21.1 | 0.013 | 0.05 |  |
|  |  | EEAA | SAP | -6.36 | -13.3 | 0.633 | 0.08 | 0.11 |
|  |  |  | ECR | -1.15 | -12.2 | 9.9 | 0.84 |  |
|  |  |  |  |  |  |  |  |  |

c. FEV_1_/FVC

| Sex | Time point | Age acceleration | Cohort | Estimate | Lower | Upper | P-value | Meta  p-value |
| --- | --- | --- | --- | --- | --- | --- | --- | --- |
|  | Baseline | AA_res_ | SAP | 0.0014 | -0.0004 | 0.0031 | 0.12 | 0.43 |
| Male |  |  | ECR | -0.0001 | -0.0014 | 0.0012 | 0.88 |  |
|  |  | IEAA | SAP | 0.0014 | -0.0004 | 0.0033 | 0.13 | 0.54 |
|  |  |  | ECR | -0.0002 | -0.0015 | 0.0011 | 0.75 |  |
|  |  | EEAA | SAP | -0.0001 | -0.001 | 0.001 | 0.86 | 0.38 |
|  |  |  | ECR | -0.0007 | -0.002 | 0.0007 | 0.33 |  |
|  | Follow-up | AA_res_ | SAP | 0.0007 | -0.001 | 0.0023 | 0.41 | 0.33 |
|  |  |  | ECR | 0.0005 | -0.0013 | 0.0023 | 0.59 |  |
|  |  | IEAA | SAP | 0.0006 | -0.0011 | 0.0023 | 0.48 | 0.54 |
|  |  |  | ECR | 0.0001 | -0.0016 | 0.0019 | 0.88 |  |
|  |  | EEAA | SAP | -0.0004 | -0.002 | 0.001 | 0.63 | 0.72 |
|  |  |  | ECR | 0.00005 | -0.002 | 0.0002 | 0.96 |  |
|  |  |  |  |  |  |  |  |  |
| Female | Baseline | AA_res_ | SAP | -0.0004 | -0.0016 | 0.0009 | 0.55 | 0.51 |
|  |  |  | ECR | -0.0002 | -0.0012 | 0.0009 | 0.71 |  |
|  |  | IEAA | SAP | -0.0007 | -0.002 | 0.0006 | 0.29 | 0.3 |
|  |  |  | ECR | -0.0003 | -0.0014 | 0.0009 | 0.64 |  |
|  |  | EEAA | SAP | -0.00006 | -0.001 | 0.001 | 0.92 | 0.31 |
|  |  |  | ECR | -0.0007 | -0.001 | 0.0004 | 0.19 |  |
|  | Follow-up | AA_res_ | SAP | -0.0006 | -0.0018 | 0.0007 | 0.33 | 0.06 |
|  |  |  | ECR | -0.001 | -0.0021 | 0.0002 | 0.09 |  |
|  |  | IEAA | SAP | -0.0004 | -0.0018 | 0.0009 | 0.53 | 0.04* |
|  |  |  | ECR | -0.0016 | -0.0029 | -0.0002 | 0.02 |  |
|  |  | EEAA | SAP | -0.0001 | -0.002 | 0.0009 | 0.84 | 0.21 |
|  |  |  | ECR | -0.001 | -0.002 | 0.00006 | 0.06 |  |
|  |  |  |  |  |  |  |  |  |
|  |  |  |  |  |  |  |  |  |

SAP = SAPALIDA, ECR = ECRHS. Lower and upper is the lower and upper ranges of 95% confidence interval of estimates.
