# Supplementary material for: Association of adult lung function with accelerated biological aging
Source: Aging (Albany NY). 2020 Jan 11;12(1):518–42. doi: 10.18632/aging.102639 (PMC6977706; doi:10.18632/aging.102639)
Supplement: Supplementary Table 7 [file aging-12-102639-s001..docx]

**Supplementary Table 7.** **Age stratified cross-sectional analysis results of association between age acceleration and lung function of females in SAPALDIA and ECRHS.**

| Age group (years) | N (Female) | N (Male) |
| --- | --- | --- |
| < 30 | 0 | 4 |
| 30-40 | 206 | 184 |
| 40-50 | 429 | 374 |
| 50-60 | 468 | 374 |
| 60-70 | 324 | 272 |
| 70-80 | 128 | 92 |
| > 80 | 3 | 8 |
| Total | 1558 | 1308 |

1. FEV_1_

|  |  |  | Female |  |  |  |  | Male |  |  |
| --- | --- | --- | --- | --- | --- | --- | --- | --- | --- | --- |
| Age acceleration | Age Group | Estimate | Lower | Upper | P-value |  | Estimate | Lower | Upper | P-value |
| AAres | 30-40 | 2.25 | -7.35 | 11.8 | 0.644 |  | 6.06 | -10.4 | 22.5 | 0.462 |
|  | 40-50 | -4.03 | -11.8 | 3.76 | 0.31 |  | 11.8 | -1.24 | 24.7 | 0.076 |
|  | 50-60 | -8.26 | -14.8 | -1.73 | 0.014* |  | -10.9 | -24.1 | 2.3 | 0.104 |
|  | 60-70 | -11.5 | -20.6 | -2.45 | 0.013* |  | -7.47 | -22 | 7.09 | 0.311 |
|  | 70-80 | 2.11 | -0.483 | 4.69 | 0.104 |  | 7.64 | -12.9 | 28.1 | 0.459 |
| IEAA | 30-40 | 1.27 | -9.31 | 11.8 | 0.813 |  | 8.23 | -9.74 | 26.2 | 0.364 |
|  | 40-50 | -7.45 | -15.6 | 0.675 | 0.072 |  | 9.29 | -3.12 | 21.6 | 0.14 |
|  | 50-60 | -10.1 | -17.8 | -2.4 | 0.01* |  | -14.7 | -27.9 | -1.41 | 0.03* |
|  | 60-70 | -11 | -20.6 | -1.42 | 0.024* |  | -0.289 | -15.5 | 15 | 0.97 |
|  | 70-80 | 1.18 | -11.3 | 13.8 | 0.851 |  | 10.9 | -9.41 | 31.1 | 0.288 |
| EEAA | 30-40 | -12.7 | -32.8 | 7.36 | 0.213 |  | -3.5 | -28.2 | 21.2 | 0.78 |
|  | 40-50 | -7.24 | -16.8 | 2.33 | 0.137 |  | -16.6 | -34.8 | 1.65 | 0.0666 |
|  | 50-60 | -2.8 | -12 | 6.33 | 0.547 |  | -5.39 | -24.8 | 14 | 0.583 |
|  | 60-70 | -1.31 | -13.3 | 10.6 | 0.828 |  | 2.54 | -17.3 | 22.3 | 0.801 |
|  | 70-80 | 16.1 | 1.51 | 30.7 | 0.03* |  | 38.5 | 8.15 | 69.1 | 0.0137 |

1. FVC

|  |  |  | Female |  |  |  |  | Male |  |  |
| --- | --- | --- | --- | --- | --- | --- | --- | --- | --- | --- |
| Age Acceleration | Age Group | Estimate | Lower | Upper | P-value |  | Estimate | Lower | Upper | P-value |
| AAres | 30-40 | 3.21 | -7.71 | 14.1 | 0.563 |  | 1.95 | -16.8 | 20.8 | 0.836 |
|  | 40-50 | -2.83 | -12.1 | 6.48 | 0.551 |  | 16.4 | -0.0569 | 32.8 | 0.051 |
|  | 50-60 | -11.5 | -20 | -3.01 | 0.008* |  | -15.7 | -32.1 | 0.785 | 0.061 |
|  | 60-70 | -11 | -21.8 | -0.133 | 0.047* |  | -11.3 | -29.7 | 6.97 | 0.222 |
|  | 70-80 | -3.43 | -18.2 | 11.3 | 0.644 |  | -3.74 | -30 | 22.3 | 0.774 |
| IEAA | 30-40 | 2.46 | -9.57 | 14.5 | 0.687 |  | 1.38 | -19.3 | 22 | 0.895 |
|  | 40-50 | -5.99 | -15.7 | 3.74 | 0.227 |  | 12.1 | -3.66 | 27.8 | 0.131 |
|  | 50-60 | -12 | -22.1 | -2.07 | 0.018* |  | -17.9 | -34.4 | -1.37 | 0.033* |
|  | 60-70 | -9.88 | -21.4 | 1.6 | 0.091 |  | -5.23 | -24.5 | 14 | 0.592 |
|  | 70-80 | 1.21 | -14.9 | 17.3 | 0.882 |  | 3.12 | -23 | 29 | 0.81 |
| EEAA | 30-40 | -10.3 | -33.2 | 12.4 | 0.37 |  | -20.5 | -48.8 | 7.72 | 0.152 |
|  | 40-50 | -5.95 | -17.4 | 5.51 | 0.308 |  | -5.36 | -27.9 | 17.2 | 0.638 |
|  | 50-60 | -3.9 | -15.5 | 7.76 | 0.511 |  | 0.931 | -23.2 | 25.1 | 0.939 |
|  | 60-70 | 0.162 | -14.1 | 14.4 | 0.982 |  | 0.155 | -24.7 | 25 | 0.99 |
|  | 70-80 | 12.8 | -5.43 | 31.1 | 0.167 |  | 45.6 | 7.13 | 84.2 | 0.021* |

1. FEV_1_/FVC

|  |  |  | Female |  |  |  |  | Male |  |  |
| --- | --- | --- | --- | --- | --- | --- | --- | --- | --- | --- |
| Age Acceleration | Age Group | Estimate | Lower | Upper | P-value |  | Estimate | Lower | Upper | P-value |
| AAres | 30-40 | -9.2E-05 | -0.003 | 0.001 | 0.9 |  | 0.0004 | -0.001 | 0.002 | 0.633 |
|  | 40-50 | -0.00001 | -0.004 | 0.004 | 0.995 |  | 0.002 | -0.003 | 0.006 | 0.386 |
|  | 50-60 | -0.002 | -0.004 | 0.001 | 0.297 |  | 0.001 | -0.003 | 0.005 | 0.594 |
|  | 60-70 | -0.0002 | -0.002 | 0.002 | 0.797 |  | 0.001 | -0.001 | 0.004 | 0.256 |
|  | 70-80 | 0.001 | -0.001 | 0.003 | 0.272 |  | 0.001 | -0.002 | 0.004 | 0.392 |
| IEAA | 30-40 | -1.6E-06 | -0.002 | 0.001 | 0.998 |  | 0.0009 | -0.001 | 0.003 | 0.361 |
|  | 40-50 | 0.0002 | -0.003 | 0.004 | 0.904 |  | 0.002 | -0.002 | 0.007 | 0.315 |
|  | 50-60 | -0.0008 | -0.004 | 0.003 | 0.682 |  | -0.0006 | -0.005 | 0.004 | 0.783 |
|  | 60-70 | -0.0006 | -0.002 | 0.001 | 0.577 |  | 0.002 | -0.0005 | 0.005 | 0.104 |
|  | 70-80 | 0.002 | -0.0006 | 0.004 | 0.149 |  | 0.001 | -0.002 | 0.004 | 0.498 |
| EEAA | 30-40 | -0.0002 | -0.003 | 0.003 | 0.912 |  | 0.002 | -0.0008 | 0.005 | 0.152 |
|  | 40-50 | -0.002 | -0.006 | 0.002 | 0.356 |  | 0.006 | -0.0003 | 0.012 | 0.063 |
|  | 50-60 | 0.0005 | -0.004 | 0.004 | 0.836 |  | -0.003 | -0.009 | 0.003 | 0.305 |
|  | 60-70 | 0.001 | -0.001 | 0.004 | 0.288 |  | 0.003 | -0.0002 | 0.006 | 0.062 |
|  | 70-80 | 0.002 | -0.0009 | 0.005 | 0.198 |  | 0.002 | -0.003 | 0.006 | 0.478 |

Lower and upper is the lower and upper ranges of 95% confidence interval of estimates.
